# Differences in Game Dynamics between High-Level Volleyball and Beach Volleyball Matches

**DOI:** 10.3390/jfmk9010028

**Published:** 2024-01-31

**Authors:** Tomislav Đurković, Domagoj Babok, Tomica Rešetar

**Affiliations:** Faculty of Kinesiology, University of Zagreb, 10000 Zagreb, Croatiatomica.resetar@kif.hr (T.R.)

**Keywords:** team sports, performance, match analysis, temporal patterns

## Abstract

The aim of this research is to verify whether there is a difference in the average duration of the active and passive phases of the game between volleyball and beach volleyball. A total of 2392 active and passive phases were measured during high-level volleyball and beach volleyball matches for males. Matches were played by four teams (53 players) in indoor volleyball (age 29 ± 4.94 years) and five teams (10 players) in beach volleyball (age 28.27 ± 6.64 years). Possible differences were evaluated using the Mann–Whitney U test. The average duration of the active phase in volleyball is 5.55 s ± 4.38 s. In beach volleyball, the active phase lasts marginally longer, at 6.00 s ± 3.44 s. The average duration of the passive phase in volleyball is 35.27 s ± 25.96 s and it lasts longer than the average passive phase in beach volleyball at 33.82 s ± 22.98 s. The Mann–Whitney U test showed a statistically significant difference (*p* = 0.00) between the active phases in volleyball (Md = 3.53, n = 727) and beach volleyball (Md = 3.43, n = 484), U = 140770.00, z = −5.90 with small effect according to Cohen’s criterion (r = 0.14). The Mann–Whitney U test (U = 160773.00, z = −1.10) showed no statistically significant difference in the average duration of the passive phases at volleyball and beach volleyball. This research determined that there is a statistically significant difference in the average duration of the active phase between volleyball and beach volleyball. The new insights gained in this research can support a more precise programming of training intensity in high-level volleyball and beach volleyball.

## 1. Introduction

Volleyball and beach volleyball are remarkably similar; however, in view of the rules of the game and the different playing surface, they are regarded as two different sports. Volleyball is a part of the competitive Olympic program since 1964 (Tokyo, Japan), while beach volleyball joined 22 years later, in 1996 (Atlanta/USA) [1]. Specific features of volleyball and beach volleyball are present in terms of the number of players, size of the playing court, specific rules, and technical-tactical demands [2,3]. The details are listed in Table 1.

According to the data listed in Table 1, it is possible to conclude that a player in beach volleyball covers more surface area, 32 m^2^, while a player in volleyball covers 13.5 m^2^. Beach volleyball players are moving on the sand which must be minimally 40 cm deep [3]. That is more complex and energetically more demanding. Energy consumption during walking or running on sand is about 20% higher than on a hard surface [4,5,6,7,8] and matches are played in more difficult conditions that include harsh sun, wind, and even rain (until the moment where weather conditions present harm to players’ health). Beach volleyball players must be universally skilled (perform all techniques equally well), while volleyball players are specialized in specific playing tasks. The rules of the game in beach volleyball [3] are formed in such a way to prevent winning points easily, as well as to prolong the active phase of the game as much as possible. Therefore, when setting the ball with the overhand pass, the rotation of the volleyball is almost not allowed, so most players set the ball with the underhand pass (the overhand pass allows for easier precision when setting the ball for a smash, as the opponent has less time available than when setting the ball with the lower, underhand pass). In beach volleyball, it is not allowed to “attack” with the overhand pass (besides in very rare situations) as this would enable easy winning points. The role of the coach in the two sports mentioned is also entirely different. In volleyball, the players are actively guided by the coach during the game, while such practice in beach volleyball is forbidden. High-level players in volleyball and beach volleyball must have excellent physical conditioning preparation. If one player is not in optimal shape, five players (volleyball) can more easily compensate for that deficiency than one (beach volleyball). Training and competing on sand surfaces cause a lower risk of musculature damage than training and competing on hard surfaces [9].

The average duration of a match in volleyball is around 100 min [10,11], and in beach volleyball it is around 51 min, including set intervals [12,13]. The measured temporal patterns are as follows, an average active phase of the game in top-level volleyball lasts 5.54 s [14], while in beach volleyball it lasts 5.82 s [13]. The average measured active phase of the game, without “pseudo-rallies” (aces and service faults) in volleyball, lasts 7.11 s [14], whereas in beach volleyball the average phase lasts 6.62 s [13]. Active phases of the game last longer in correlation with age categories switching to older ones [15]. Regarding the duration of the active phase of the game, the conclusion can be made that both players of indoor and beach volleyball must have a well-developed anaerobe-phosphagen energetic system, as well as the anaerobic glycolytic system, as certain active phases can last even longer than 25 s, or even up to 40 s. The base for these two energetic systems is certainly the aerobic system, whose high level is provided with higher-intensity training, lasting between 90 and 120 min. The values of VO_2max_ for the group of volleyball players on the national teams’ range between 55 and 60 mL kg^−1^ min^−1^ [16,17,18,19,20,21,22]. The percentage of “flying ball”, i.e., the overall percentage of all added up active phases divided with the overall match time duration (excluding intervals between sets), goes in favor of beach volleyball with 19.50% [13], in comparison to 15.62% [14] for volleyball. In beach volleyball there are fewer ace serves; however, there are also less service faults (14.65%) [13], as opposed to volleyball where that percentage is as high as 23.44% [14]. Volleyball has a higher number of average ball contacts (6.76) [14], while that number in beach volleyball is 5.81 [13]. In some older studies, the ratio between active and passive phases in volleyball (rally point system) is 1:2.4 [23] or 1:3 [24]. In recent research, this ratio ranges from 1:5.52 to 1:5.81 [25,26]. In beach volleyball, the mentioned ratio is more favorable for competitors, as it is 1:4 [27]. Upon considering the listed specificities, differences in the average duration of active and passive phases of the game between these two sports are possible. The subject of this research is precisely the average duration of active and passive phases in volleyball and beach volleyball in the men’s senior category, on a sample of top volleyball competitors. Upon measuring the time elapsed in the active and passive phases of the game, average values shall be calculated and compared between the mentioned two sports. The aim of this research is to determine if there is a statistically significant difference in the duration of the active and passive phases of the game between volleyball and beach volleyball on a sample of high-level male volleyball players. We hypothesize that, due to the already mentioned specificities arising from the rules and tactics of these two games, the average duration of the active and passive phases could be statistically significantly different. Information about the time duration of active and passive phases in these two sports, and the possible difference between them could be used in the field of programming specific training. Based on the obtained results, the coaches will be able to optimally program (dosage) the intensity of the training load with the aim of increasing the specific fitness preparation and situational efficiency of the players.

## 2. Materials and Methods

### 2.1. Participants

Measurements were completed on a sample of 4 high-level club teams in volleyball and 5 high-level teams (pairs) in beach volleyball. In indoor volleyball, 53 players were 29 ± 4.94 years old, 197.64 ± 9.23 cm tall, and weighed 89.19 ± 13.12 kg, while in beach volleyball, 10 players were 28.27 ± 6.64 years old, 197.30 ± 7.33 cm tall, and weighed 87.60 5.27 kg. Demographic data for players were downloaded from the official website of International Volleyball Federation (Fédération Internationale de Volleyball). The volleyball matches were selected from the matches played during 2019 FIVB Volleyball Club World Championship for Men, held in Betim (Portugal), as well as the matches from the 2019 Men’s Beach Volleyball World Championship, held in Hamburg (Germany). A total of 10 matches—5 volleyball matches and 5 beach volleyball matches—were selected for the purposes of this research. Given the fact that the entities are represented by the points played, a larger number of matches was not necessary. A total of 2392 active and passive phases were measured during matches.

### 2.2. Variables and Equipment

In this research, the entities are the played points, i.e., the active and passive phase of each of the played points. Thus, after each played point, one entity representing the duration of the active phase and one entity representing the duration of the passive phase is obtained. During the indoor and beach volleyball competitions, 4 variables were monitored, namely:

ACPH_BEACH (Duration of active phase in beach volleyball)—The duration of the active phase in beach volleyball starts with the server contacting the ball and lasts until the moment the ball touches the ground, or if the player commits a foul or is penalized, which precedes the whistle of one of the referees.

PASPH_BEACH (Duration of passive phase in beach volleyball)—The duration of the passive phase in beach volleyball starts at the moment the previous active phase ends and lasts until the beginning of the following active phase. It should be emphasized that time-outs and referee explanations were also measured and classified as passive phases, while the time needed to change sides between sets was not.

ACPH_VOLLEY (Duration of active phase in volleyball)—The duration of the active phase in volleyball starts with the server contacting the ball and lasts until the moment the ball touches the ground, or if the player commits a foul or is penalized, which precedes the whistle of one of the referees.

PASPH_VOLLEY (Duration of passive phase in volleyball)—The duration of the passive phase in volleyball starts at the moment the previous active phase ends and lasts until the beginning of the following active phase. It should be emphasized that time-outs, referee explanations, and the time required for player substitutions were measured and classified as passive phases, while the time required for changing sides between sets was not.

Selected matches used in this research were recorded via official cameras during the duration of the competition.

### 2.3. Protocol of Investigation

The pre-recorded matches were downloaded from the official channel of the International Volleyball Federation (FIVB Channel). The duration of each active and passive phase was measured using Windows Media Player software 12.0.9600.17031.

The process of measuring the active and passive phases of the game was carried out by an experienced kinesiologist, who is a former volleyball and beach volleyball player and is one of the authors of this research.

For the active phase of the game, the measurer starts the measurement when the server touches the ball and stops at the moment the ball touches the ground, or if the player commits a foul or is penalized, which precedes the whistle of one of the referees. The measured time represents the duration of the active phase. The measured time is expressed in seconds.

For the passive phase, the measurer starts the measurement at the end of the previous active phase and stops at the beginning of the next active phase. The measured time represents the duration of the passive phase. The measured time is expressed in seconds.

In the previously described way, the measured values of the active and passive phases were entered into an ad hoc prepared Excel table.

### 2.4. Statistical Analysis

With the use of a G*power program 3.1.9.7 (University of Dusseldorf, Dusseldorf, Germany), the sample size was calculated (n = 102) that was needed for the testing procedure with a statistical significance of *p* ≤ 0.05; statistical power 0.80; effect size 0.3; and two groups. Statistical package Statistica, version 13.5.0.17 (TIBCO Software Inc., Palo Alto, CA, USA) was used for data analysis. For all measurement variables, descriptive parameters (arithmetic mean, minimum and maximum values, and standard deviation) were calculated. Normality of data distribution was tested by the Kolmogorov–Smirnov test. Since all measured variables deviated from normal distribution, the Mann–Whitney U test was used for verifying statistical significance. In addition, the Spearman’s correlation coefficient was calculated between the average value of the active and passive phase in volleyball and beach volleyball. Specifically, both for volleyball and beach volleyball, the “flying ball” coefficient (percentage of active game) and work-to-rest ratio was also calculated. The “flying ball” coefficient was obtained by dividing the average value of the active phase with the average value of the passive phase and work-to-rest was obtained by the active phase divided by the passive phase values. Overall, 11.50% (165) of the active and passive phases were re-analyzed for volleyball and 11.50% (110) for beach volleyball, surpassing the reference value of 10% [28]. Cohens kappa ranged from 0.81 to 0.85 for inter-observer reliability, and between 0.80 and 0.87 for intra-observer reliability, surpassing the suggested value of 0.75 [29].

## 3. Results

The data indicated in Table 2 present descriptive parameters in matches of volleyball and beach volleyball. It is evident that the average active phase lasts longer in beach volleyball (6.00 ± 3.44 s). In addition, beach volleyball players have a slightly shorter time to recover (33.82 ± 22.98 s). The “flying ball” coefficient is higher in beach volleyball (17.7%) than in indoor volleyball (15.5%). The average work-to-rest ratio is 1:6.35 s for volleyball and 1:5.63 s for beach volleyball.

The Mann–Whitney U test for the duration of active and passive phases in volleyball and beach volleyball (Table 3) registered a statistically significant difference (*p* = 0.00) between the duration of active phases played in volleyball matches (Md = 3.53, n = 727) when compared to active phases played in beach volleyball matches (Md = 3.43, n = 484), U = 140770.00, z = −5.90, with a small to medium impact, according to the Cohen criterion (r = 0.14). A statistically significant difference for the duration of passive phases was not found.

Spearman’s correlations test revealed positive and statistically significant correlations (*p* = 0.00) with medium-strong influence between the duration of active and passive phases both in volleyball (rho = 0.3) and in beach volleyball (rho = 0.29).

## 4. Discussion

The present study had one aim—to determine if there is a statistically significant difference in the duration of the active and passive phases of the game between volleyball and beach volleyball on a sample of high-level male volleyball players. Due to the specific rules and tactics of these two games, the hypothesis was that the average duration of the active and passive phases could be statistically significantly different.

A significant difference was registered between the average durations of active phases.

For volleyball, a total of 1438 entities were measured in the sports hall (727 active and 711 passive phases). The shortest active phase lasted 0.75 s, while the longest one lasted 30.19 s. The arithmetic mean for active phases was 5.55 ± 4.38 s, which is very similar to the results of the previous research on male senior volleyball players [14,25,26,30]. In beach volleyball, a total of 954 entities were measured (484 active and 470 passive phases). The shortest active phase lasted 0.66 s, while the longest was 34.33 s. The arithmetic mean for the active phases is 6.00 ± 3.44, which is slightly longer than in the research obtained on the top sample of senior male volleyball players [13] shown in Table 1.

The average active phase lasts significantly longer in beach volleyball than in volleyball. Upon considering the weather conditions, playing surface, number of players, and the information that the average active phase is statistically significantly longer, it is logical that caloric consumption in beach volleyball is higher, up to 25%, than that in volleyball, which has been confirmed in practice [31].

Significant differences in the average duration of the passive phases were not found. The shortest passive phase in volleyball lasted 14.46 s, whereas the longest was 385.69 s. The arithmetic mean for the passive phases in volleyball was 35.27 ± 25.96. The shortest passive phase in beach volleyball lasted 13.83 s, whereas the longest was 259.28 s. The arithmetic mean for passive phases in beach volleyball was 33.82 ± 22.98. The average passive phase lasts longer in indoor volleyball (not statistically relevant), which indicates that after an averagely longer active phase, beach volleyball players have less time to recover until the following point. With regard to the significantly longer active phase and shorter passive phase, it can be assumed that beach volleyball players should have a higher level of anaerobic capacity when compared to volleyball players, and thus indirectly also have a higher level of aerobic capacity as well. However, the results of VO2 max research show that both indoor [16,17,18,19,20] and beach volleyball [7,32] values range from 55 to 65 mL kg^−1^ min^−1^.

The coefficient of volleyball “flying ball” was 0.157, which means that in the overall duration of volleyball matches, the ball was in the air 15.7% of the time, which is almost identical to the results of the FIVB research on top male senior volleyball players [14] and confirms the top quality of the tested sample.

The coefficient of beach volleyball “flying ball” was 0.177, which means that in the total duration of beach volleyball matches, the ball was in the air 17.7% of the time, which is slightly less than 19.50%, obtained on a similar, superior sample of men’s senior beach volleyball players [13].

The average ratio of work and rest in volleyball is 1:6.35, which is closer to the results of more recent research, which range from 1:5.52 to 1:5.81 [25,26]. The slightly higher ratio in favor of the passive phase is likely caused by the difference in data collection methodology. Namely, that research did not count the time required for time-outs, referee explanations, and player substitutions in the duration of the passive phases, which should be considered incorrect as the competitors have that time to recover between the two active phases [26].

The average ratio of work and rest in beach volleyball is 1:5.63, which is a slightly lower ratio than for volleyball. This is also likely caused by the difference in data collection methodology. Namely, that research did not count the time required for time-outs, referee explanations, and player substitutions in the duration of the passive phases, which should be considered incorrect as the competitors have that time to recover between the two active phases. The results of the previous research on similar samples are somewhat smaller, 1:3.5 [33] and 1:4.57 [27], which may also be related to the already mentioned difference in measurement methodology.

Positive and statistically significant correlations between the duration of active and passive phases both in volleyball and in beach volleyball were revealed. In simpler terms, if the active phase does not last long, then the passive phase is also shorter. If the active phase lasts longer, the passive phase shall also last longer. The mentioned phenomenon is not anticipated in the rules; however, in practice it is evident [26] that volleyball players consciously take a longer break after a longer active phase, which leads to exhaustion of the nervous as well as the metabolic system [34]. The referee’s intuition after a long, exhausting point allows for a slightly longer break is also helpful. The conducted research also determined there is a moderately strong positive correlation between the duration of active and passive phases, i.e., if the active phase of the game is longer, then the passive phase of the game shall also be somewhat longer. The obtained data can be used for a more precise determination of the load (intervals of work and rest) during volleyball training. This is especially important in the preparatory part of the season in the stage of specific preparation of volleyball players. Such training can be realized individually, through the repetition of a certain technical element using the “synthetic method”, where a certain technical-tactical element is performed through successive repetitions with smaller time breaks (simulation of the competitive rhythm—“work to rest ratio”), or the “global method” i.e., by playing 6 vs. 6 with specific tactical tasks and additional throwing of balls after the end of the previous active phase (so-called “Wash” exercises). The goal of throwing in additional balls is to shorten the passive phase to achieve better adaptation to the upcoming competitive loads.

### Limitations

The results obtained in this research were collected at two tournaments where competitions were held at the highest level in a men’s senior competition. At the World Club Indoor Volleyball Championship, ten games were played, and five of them were analyzed. At the Beach Volleyball World Championship, fifty-five games were played, and five of them were also analyzed. The competing teams represent the top of world quality, so the differences between them are marginal and the testing sample can be considered very homogeneous. Although the limit of the research could be considered, the fact that not all matches in the mentioned competitions were processed, the conclusions presented are based on a large number of measured active and passive phases (2392), and the obtained data can be considered relevant for drawing conclusions about the characteristics of competitions at the high-level.

## 5. Conclusions

This research revealed a statistically significant difference in the average duration of the active phase. The average active phase lasts longer in beach volleyball than in volleyball. A statistically significant difference for the duration of passive phases was not found. Beach volleyball players have a slightly shorter time to recover, which was not deemed statistically significant. A higher “flying ball” coefficient and lower average work-to-rest ratio puts beach volleyball players in front of more demanding energy challenges. The obtained data—temporal patterns—can be used by coaches and athletes in developing physical conditioning, i.e., aerobic, and anaerobic, capacities. Data on the average duration of the active and passive phase allow coaches to precisely define periods of play and periods of recovery in their training process to ensure players are prepared for situational conditions. This type of research could be conducted with different age categories, and thus compare the average duration of active and passive phases in the point both in youth (U17) and junior (U19) players.

## Figures and Tables

**Table 1:** Specific features of volleyball and beach volleyball.

	Volleyball	Beach Volleyball
Number of players on court	6	2
Dimensions of the court	18 m × 9 m	16 m × 8 m
To win	3 sets	2 sets
Set/points	25	21
Tie break	15 points to win	15 points to win
Surface (FIVB competitions)	Wooden or synthetic	Sand
Minimum temperature	16 °C	Not defined: The weather must not present any danger of injury to the players
Maximum temperature	26 °C	Not defined: The weather must not present any danger of injury to the players
Coaching during game	Yes	No/Forbidden to receive external assistance or coaching during a match
Playing—serve receive	Underhand and overhand passing	Underhand passing
Playing—attacking	Player can attack with overhand pass	It is forbidden to attack with overhand pass
Ball/material	Made of a flexible material (leather, synthetic leather, or similar)	Made of a flexible material (leather, synthetic leather, or similar) which does not absorb moisture, i.e., more suitable to outdoor conditions since matches can be played when it is raining
Ball/dimensions/circumference	65–67 cm	66–68 cm
Ball/dimensions/weight	260–280 g	260–280 g
Ball/dimensions/inside pressure	0.3–0.325 kg/cm^2^	0.175–0.225 kg/cm^2^
Average match duration	100 min	51 min
Average rally duration	5.54 s	5.82 s
Average rally duration without pseudo-rallies	7.11 s	6.62 s
“Flying ball” * (excluding set intervals)	15.62%	19.50%
Portion of “Pseudo-rallies” ** (ace or service fault, about 1 s)	25.63%	14.65%
Average amount of ball contacts during one rally	6.76	5.81

* “Flying ball”—portion of active playing time during one volleyball game. ** “Pseudo-rallies”—rallies finished with ace or service fault.

**Table 2:** Descriptive parameters of measured variables.

Variable	n	Min	Max	Mean ± SD	“Flying Ball”	Work-to-Rest Ratio
ACPH_VOLLEY	727	0.75	30.19	5.55 ± 4.38	15.7%	1:6.35
PASPH_VOLLEY	711	14.46	385.69	35.27 ± 25.96		
ACPH_BEACH	484	0.66	34.33	6.00 ± 3.44	17.7%	1:5.63
PASPH_BEACH	470	13.83	259.28	33.82 ± 22.98		

n (number of entities), Min (minimum value), Max (maximum value), Mean (arithmetic mean), SD (standard deviation).

**Table 3:** Results of the Mann–Whitney U test for the active and passive phases of the game in volleyball and beach volleyball.

	Active Phase	Passive Phase
MWU	140,770.00	160,773.00
WW	405,398.00	271,458.00
Z	−5.90	−1.10
*p*	0.00 *	0.27

MWU—value of Mann–Whitney U test; WW—value of Wilcoxon W; Z—Z value of test; *p*—level of statistical significance of test; * marks statistically significant difference (*p* < 0.05).

## Data Availability

The data presented in this study are available on request from the corresponding author. The data are not publicly available due to its huge size and participants privacy protection.
